# Benchmarking Spectroscopic Techniques Combined with Machine Learning to Study Oak Barrels for Wine Ageing

**DOI:** 10.3390/bios12040227

**Published:** 2022-04-09

**Authors:** Tatevik Chalyan, Indy Magnus, Maria Konstantaki, Stavros Pissadakis, Zacharias Diamantakis, Hugo Thienpont, Heidi Ottevaere

**Affiliations:** 1 Brussels Photonics (B-PHOT), Department of Applied Physics and Photonics, Vrije Universiteit Brussel, Pleinlaan 2, B-1050 Brussels, Belgium; indy.magnus@vub.be (I.M.); hugo.thienpont@vub.be (H.T.); heidi.ottevaere@vub.be (H.O.); 2 Institute of Electronic Structure and Laser (IESL), Foundation for Research and Technology-Hellas (FORTH), 70013 Heraklion, Greece; mkonst@iesl.forth.gr (M.K.); pissas@iesl.forth.gr (S.P.); 3 Winemakers’ Association of the Department of Heraklion—Wines of Crete, Archimidous 1 & Ikarou St., 71601 Heraklion, Greece; zd@diamantakiswines.gr

**Keywords:** wine ageing, oak barrel, lignin, tannin, Raman spectroscopy, fluorescence spectroscopy, reflectance spectroscopy, chemometrics

## Abstract

Due to its physical, chemical, and structural properties, oakwood is widely used in the production of barrels for wine ageing. When in contact with the wine, oak continuously releases aromatic compounds such as lignin, tannin, and cellulose to the liquid. Due to the release process, oak loses its characteristic aromatic compounds in time; hence, the flavour that it gives to the enclosed wine decreases for repeated wine refills and a barrel replacement is required. Currently, the estimation of the maximum number of refills is empirical and its underestimation or overestimation can impose unnecessary costs and impair the quality of the wine. Therefore, there is a clear need to quantify the presence of the aforementioned aromatic compounds in an oak barrel prior to a refill. This work constitutes a study to examine noninvasive optical biosensing techniques for the characterization of an oak barrel used in wine ageing, towards the development of a model to unveil its lifespan without inducing structural damage. Spectroscopic diagnostic techniques, such as reflectance, fluorescence, and Raman scattering measurements are employed to assess the change in the chemical composition of the oakwood barrel (tannin and lignin presence) and its dependence on repeated refills. To our knowledge, this is the first time that we present a benchmarking study of oak barrel ageing characteristics through spectroscopic methods for the wine industry. The spectroscopic data are processed using standard chemometric techniques, such as Linear Discriminant Analysis and Partial Least Squares Discriminant Analysis. Results of a study of fresh, one-time-used, and two-times-used oak barrel samples demonstrate that reflectance spectroscopy can be a valuable tool for the characterization of oak barrels. Moreover, reflectance spectroscopy has demonstrated the most accurate classification performance. The highest accuracy has been obtained by a Partial Least Squares Discriminant Analysis model that has been able to classify all the oakwood samples from the barrels with >99% accuracy. These preliminary results pave a way for the application of cost-effective and non-invasive biosensing techniques based on reflectance spectroscopy for oak barrels assessment.

## 1. Introduction

Wine production is a natural agricultural process integral to European life and culture. The wineries in the European Union are world leaders, responsible for more than 60% of the worldwide wine production with 50% of global consumption, based on the EU reports on the global wine sector [1]. However, the European wine sector is rather fragmented and winemaking remains mainly in the hands of small- to medium-sized producers. In the last decades, the evolution of the wine market in terms of demand, supply and competition has prompted the establishment of wine makers’ clusters in different regions across Europe.

As an agricultural product, wine quality highly depends on a set of parameters related to the natural influences rather than the ones added to manufacturing recipes. Wine quality and traits change from one year to the next and depend drastically on parameters such as soil and weather conditions, characteristics of the harvest, grape quality, fermentation environment, and the ageing process [2]. The ageing process, in particular, is a decisive factor that gives the wine its unique mouthfeel and bouquet character. During ageing, wine is usually placed into oak barrels since oakwood exhibits certain wetting and component release properties making it especially suitable for wine barrels [3]. During the ageing process, a series of reactions take place, resulting in changes in the chemical composition and organoleptic properties of wine affecting its final quality [4]. Apart from its favourable physical properties, such as being bendable and not prone to leakage, oakwood itself plays an important role in the ageing of wine. Oak allows mid oxidization of the enclosed wine and releases tannins and flavour compounds, such as vanillin and oak lactone isomers that complement its flavour profile [5,6]. These are the compounds that interact with the wine, thus giving a desirable flavour to the latter. Although more than 600 different oak species are found globally, only 3 species are suitable for making wine barrels [7]. Furthermore, it is estimated that, currently, around 25% of the wines produced in EU is aged in oak casks. The limited availability of oak along with the demanding barrel fabrication process increases the cost of oak barrels. The price of a new 225 L oak barrel can vary between USD 400 and USD 1000 depending on its wood origin (American Oak or French Oak) and crafting [8,9]. Furthermore, after a number of refills of the barrels with wine, the barrels become neutral and do not enrich the wine with sufficient aroma and flavour. However, the discarded barrels can still be used for other purposes. For example, adding fresh oakwood chips to the wine stored in old barrels can serve as a good alternative for winemakers. Studies have shown that the use of oak chips can be considered a good choice for producing short-aged wines. Moreover, it allows reusing used barrels in good sanitary conditions, although the overall quality is better in wines aged in new oak barrels [10]. Therefore, an investigation through reliable and non-destructive techniques, to determine whether an oak barrel still contains necessary chemical compounds needed in wine ageing, is in high demand by winemakers.

Historically, wine tasting has always been the most common way to characterize wine. However, this approach is qualitative and does not allow accurate planning of the ageing process at early stages. In this work, in order to characterize the barrels between refills, we introduce spectroscopic techniques such as reflectance, fluorescence, and Raman spectroscopy. These spectroscopic methods are used to investigate the quantitative correlation between chemical compounds present in oakwood, such as tannin and lignin, responsible for the wine’s flavour, and the number of wine refills. The use of Raman spectroscopy in the detection of tannin changes in agricultural products is a known technique [11]. The concentration of tannin derived from the calibration curve of the Raman signal intensity at 650 cm${}^{-1}$, 1357 cm${}^{-1}$, and 1590 cm${}^{-1}$ can indicate the level of maturity of pomegranate. Moreover, studies on lignin monitoring with Raman spectroscopy are reported [12]. In addition, due to the strong autofluorescence abilities of lignin, fluorescence spectroscopy is often the favoured technique to study this chemical compound [13,14]. Using excitation wavelengths in the ultraviolet (UV) range, such as 300–330 nm, fluorescence emission is observed in the 370–475 nm range with a maximum at around 385 nm. Finally, near-infrared (NIR) spectroscopy is widely used for oakwood classification. The type of oak can be derived by applying data processing algorithms on NIR spectroscopic data [15]. Furthermore, chemometric techniques for data processing are becoming more popular in various applications, such as environmental monitoring [16], food research [17], and biophotonics [18], where classification of the target analytes is required. Implementation of chemometric algorithms ease the classification process in wood species and therefore it is a valid tool in the study of oak barrel properties. The spectroscopic data in this work, therefore, is analyzed through standard chemometric techniques, such as Linear Discriminant Analysis (LDA) and Partial Least Squares Discriminant Analysis (PLS-DA).

To our knowledge, the current study combines for the first time reflectance, fluorescence, and Raman spectroscopy with data processing algorithms to assess oak barrels used repeatedly in wine ageing. We correlate the optical signal indicating the presence of lignin and tannin with the number of refills the barrels undergo. This study paves a way for further development of biosensors based on spectroscopic techniques combined with machine learning algorithms for non-invasive methods of revealing the quality of oak barrels in wine ageing.

## 2. Materials and Methods

In this work, we report preliminary results of the spectroscopic study on three different barrel pieces from French oak: fresh (no prior contact with wine), one-time-used (1 wine filling) and two-times-used (2 wine refills). All samples are from barrels of Diamantakis Winery, a member of the Wines of Crete network which was established in 2006 [19] and represents more than 95% of the bottled wine production on the island of Crete. The barrels have been manufactured in the same cooperage firm (Tonnellerie Quintessence, France). Moreover, to avoid additional uncertainties in the results, the oakwood samples have been taken from barrels used in the ageing of the same white wine variety (Vidiano, a local Cretan variety) for a 3-month period per ageing cycle. Due to the fact that the aforementioned oakwood samples have been obtained through dismantling the barrels, this study is limited to three samples to minimize the cost of the performed laboratory measurements. To compare different spectroscopic techniques, we have performed reflectance, fluorescence, and Raman spectroscopy measurements. In Section 2, the experimental setups and the obtained raw data are presented. Next, data processing algorithms are implemented on the shown raw data. In Section 3, the results of these data processing algorithms are discussed in more detail.

### 2.1. Reflectance Spectroscopy

**Figure 1:**
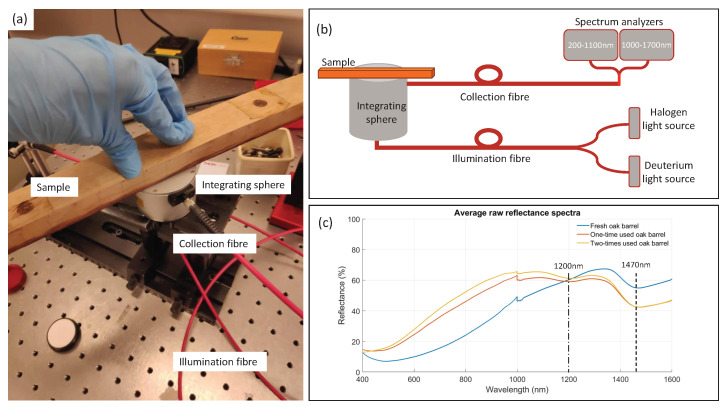
Measurement setup for reflectance spectroscopy. (**a**) Using a setup consisting of broadband light sources, optical fibres, an integrating sphere, and spectrum analyzer, the reflectance spectrum of the oak samples can be accurately measured. (**b**) Schematic overview of the experimental setup. (**c**) Raw reflectance spectra of three oakwood samples. A jump in spectra at 1000 nm is due to the stitching of two spectrum analyzers. The peaks at 1200 nm and 1470 nm indicate the characteristic bonds for cellulose, water, and lignin.

To perform the reflectance spectroscopy measurements, a reflection-integrating sphere (AvaSphere-30 of Avantes) was used. The integrating sphere collected all the reflected light coming from the sample, independently of the surface scattering or reflection angle (Figure 1). A halogen and deuterium light source were pigtailed and illuminated the sample with broadband light covering the UV, visible, and NIR spectral regions (200–2500 nm). The light source was coupled into the integrating sphere with an optical fibre (FC-UVIR600-2 of Avantes). A collimating lens (COL-UV/VIS of Avantes) was used to ensure an efficient (>90%) light coupling to the optical fibre and integrating sphere. The sample was illuminated under an angle of 8°, while all the reflected light was captured inside the sphere. The value for the angle was chosen with the purpose to avoid back-reflections. After reflecting on the inner walls of the sphere, coated with 98% reflective Spectralon, another optical fibre (FCB-UVIR600-2 fibre of Avantes) directed the light towards a broadband spectrum analyzer. The spectrum analyzer was able to measure both UV, visible, and near-infrared light. It consisted of the Avantes AvaSpec3684 spectrometer (200–1100 nm, 1.4 nm resolution) and the Avantes AvaSpec256 spectrometer (1000–1700 nm, 4 nm resolution). The Avasoft 8 software was used to calculate the reflectance spectra, as the ratio of the reflected light of the sample and the reflected light of a white (99.9% reflectivity) diffuse Spectralon tile, both corrected for the dark spectrum. The settings for the UV/VIS reflectance spectrum were an integration time of 3.7 s, 2 averages, and 37 smoothing pixels, while for the NIR spectrum, an integration time of 2.1 s, 2 averages, and 12 smoothing pixels were used. The integration time was chosen to have the largest dynamic range while avoiding saturation. The chosen numbers of smoothing pixels depend on the spectrometer slit size and the detector pixel size.

### 2.2. Fluorescence Spectroscopy

**Figure 2:**
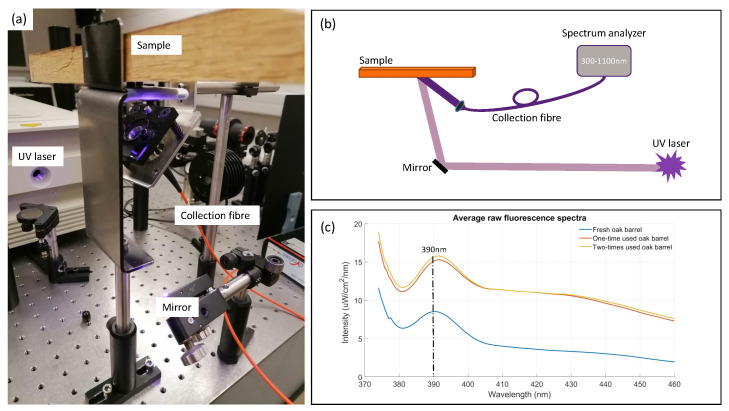
Fluorescence spectroscopy measurement setup. (**a**) Experimental setup. An excitation laser, detection fibre, and spectrum analyzer are used to capture the weak fluorescence intensity of the oak samples. (**b**) Schematic overview. (**c**) Raw fluorescence spectra of three oak samples. The peak at 390 nm represents the autofluorescence emission maximum of lignin.

The fluorescence spectroscopy measurements were performed with an excitation light source at 355 nm (Figure 2a). Although for the fluorescence measurements shorter wavelengths were favourable, 355 nm was the shortest one available in the laboratory. This wavelength was achieved by passing the 710 nm laser pulse of a Tsunami ultrafast Ti–Sapphire oscillator through the second-harmonic generating crystal. The chosen laser power was 300 mW. This was sufficient to achieve clear fluorescence signals, while higher laser power could burn the oakwood sample. An optical mirror directed the excitation laser light towards the sample. The generated fluorescence emission was collected with a broadband detection optical fibre (UVIR600 fibre of Avantes, transmitting light between 250 nm and 2500 nm) using a collimating lens. The fibre was then connected to the AvaSpec2048 spectrum analyzer (300–1100 nm, 8 nm resolution) with a wide entrance slit (200 μm) to measure the emitted fluorescence spectra with the Avasoft 8 software. Figure 2b is a schematic representation of the setup, while Figure 2c shows the raw spectra for fresh, one-, and two-times-used oak barrel’s inner surface. A fluorescence emission maximum at around 390 nm, mainly caused by the autofluorescence of lignin present in oakwood, is observed [13]. The parameters used for the optimal fluorescence measurements were an integration time of 1 ms while averaging 100 scans over time and using 6 smoothing pixels. The number of photon counts were converted to an absolute irradiance (in µW/cm²/nm) using a known transfer function taking the sensitivity of the silicon detector and optical fibre losses into account.

### 2.3. Raman Spectroscopy

The Raman measurements were performed with a commercial InVia confocal Raman system from Renishaw [20]. The system is equipped with two lasers: DPSS laser, 50 mW at 532 nm, air-cooled and kinematic mount (includes bandpass filter for 532 nm) and Diode laser, 100 mW at 785 nm, air-cooled and kinematic mount (includes bandpass filter for 785 nm), correspondingly with 1800 L/mm and 1200 L/mm grating assemblies. The resolution of the confocal Raman spectrometer is <1 cm${}^{-1}$ and the working range of the spectrometer is from 100 cm${}^{-1}$ to 3000 cm${}^{-1}$. To change the laser power, the system is equipped by a motorized neutral density filter offering 16 different power levels from 0.00005 to 100%. The laser light was sent to the sample through the objective. The same objective was later used to collect the scattered Raman signal.

**Figure 3:**
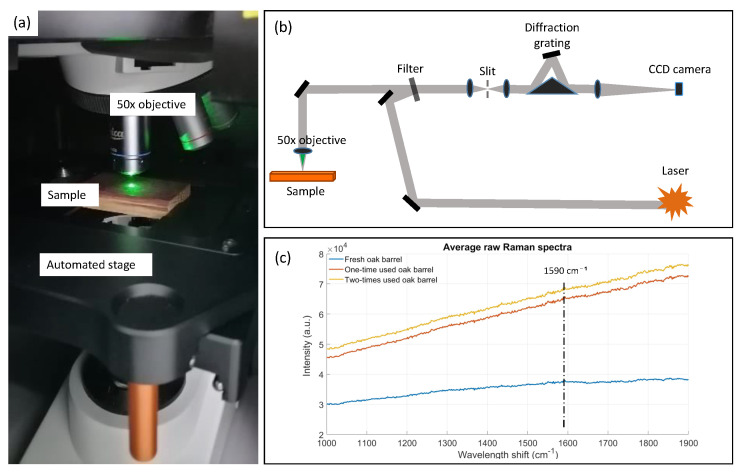
Raman spectroscopy measurement setup. (**a**) Oakwood is placed in the InVia Renishaw confocal Raman spectrometer and illuminated with 532 nm laser spot. (**b**) Schematic overview. (**c**) Raw Raman spectra of three oak samples.

The piece of wood was positioned under the Leica NPLAN 50× long working distance objective of NA = 0.75, WD 8.2 mm (Figure 3a). The fully automatized stage was used to control the height of the sample and, respectively, focus the light on the oak sample’s surface. Figure 3b represents the schematics of the setup. Raman spectra and the image of the oak surface were captured by a CCD camera sending the data to WiRE 5.3 software (Renishaw). Figure 3c represents the raw spectra of fresh, one-, and two-times-used oak barrel inner surface. One can notice that to distinguish the peak of tannin at 1590 cm${}^{-1}$ without data processing is almost impossible.

### 2.4. Chemometrics

Chemometrics can be seen as a sub-set of the data-driven machine learning field. Its goal is to extract chemical information from the measured spectroscopic data. In this work, we are mainly interested in supervised classification methods, in which a mapping from the input, i.e., the spectra, and given discrete output, i.e., the number of refills, is learned.

To remove unwanted signals and improve the classification performance, the raw spectroscopic data are usually first pre-processed. Scattering effects inside the solid samples leading to different optical path lengths for the same chemical concentration are commonly removed by using a Standard Normal Variate transformation [21]. Each spectrum is corrected by subtracting its spectral mean and dividing by its spectral standard deviation. Smoothing is done by applying a Savitsky–Golay filter, which locally replaces the spectrum by a smooth low-degree polynomial, fitted using linear least-squares [22]. By using the derivatives of the polynomial, the derivative spectrum can be found as well. This removes baseline offsets and can highlight important regions that are more difficult to identify in the original spectrum.

Principal Component Analysis (PCA) is often used to decorrelate the spectral data and transform it into a lower-dimensional space that keeps most of the variance (information) that was present in the original input signal [23]. The transformed data (scores) are found by projecting the original data onto a loading matrix. This matrix consists of the eigenvectors of the covariance matrix of the original mean-centred data, with the largest corresponding eigenvalues explaining most of the variance.

Partial Least Squares Discriminant Analysis is one of the most popular classification methods in chemometrics [24]. It is an adaptation of the Partial Least Squares regression technique, which is a linear regression applied on the projected input and output score matrices, which maximizes the covariance between input and output. By keeping only the first few scores, a regression with fewer unknowns than the number of equations can be performed, otherwise impossible on the original spectral data.

A second widely used linear classification technique is the Linear Discriminant Analysis [25]. It is a Bayesian classifier with a closed-form solution and no real parameters to tune. It assumes a multivariate Gaussian distribution, with a different mean but the same covariance, for the different classes. By maximizing the posterior probability of belonging to a certain class, given the observation, the decision boundary can be found.

## 3. Results and Discussion

The obtained spectra from the different optical measurement techniques have been analyzed by using the aforementioned classical chemometric techniques. The goal is to classify and differentiate oak barrels (classification with three classes) of the different number of refills by taking spectroscopic data from their inner walls and correlating those data with the present compounds that define wine quality.

### 3.1. Reflectance Spectroscopy

Due to the limited number of available oakwood barrel samples, multiple measurements were performed on different points of the same oakwood piece, equivalent to simulating multiple barrels with the same number of contacts with wine. One could use this assumption based on the fact that in a wine cellar the wine from different oak barrels was kept in the same conditions, i.e., the same quality, ageing, and type of oak, for the same duration of time to result in the same wine. A barrel stave was divided into three regions corresponding to the upper third (top), middle third (middle), and bottom third (bottom) parts of the barrel. More specifically, we performed 65 measurements on fresh oak: 22 on the top, 21 on the middle, and 22 on the bottom of the wood; 84 measurements with the one-time-used barrel, respectively: 28 on the top, 28 on the middle, 28 on the bottom part; and 68 measurements with the two-times-used barrel: 22 on the top, 23 on the middle, and 23 on the bottom of the wood. The number of measurements was different based on the dimensions of the small regions of the stave. All measurements were equivalently distributed horizontally over the inner part of the barrel in contact with wine shown in Figure 1.

Two characteristic absorption bands at 1200 nm and 1470 nm (Figure 1c) were considered for the analyses. The absorption at 1200 nm was mostly related to the presence of cellulose (potentially also due to lignin) in the oak (2nd overtone of CH-stretch), while the absorption at 1470 nm had contributions of cellulose (1st overtone OH-stretch), absorbed water and lignin [26,27,28,29].

To obtain the best classification performance, different types of data pre-processing have been applied to the measured spectra. Next to using the raw, unprocessed data, five pre-processing techniques that are typically used when working with (diffuse) reflectance spectroscopic data are considered here, as explained in Section 2.4: (1) Standard Normal Variate (SNV) transformation; (2) 1st derivative using a Savitsky–Golay filter of order 2 and length of 11 points; (3) 2nd derivative using a Savitsky–Golay filter of order 2 and length of 11 points; (4) SNV followed by 1st derivative; (5) SNV followed by 2nd derivative. Note that smoothing has already been done in the Avasoft 8 software by specifying a number of smoothing pixels.

**Figure 4:**
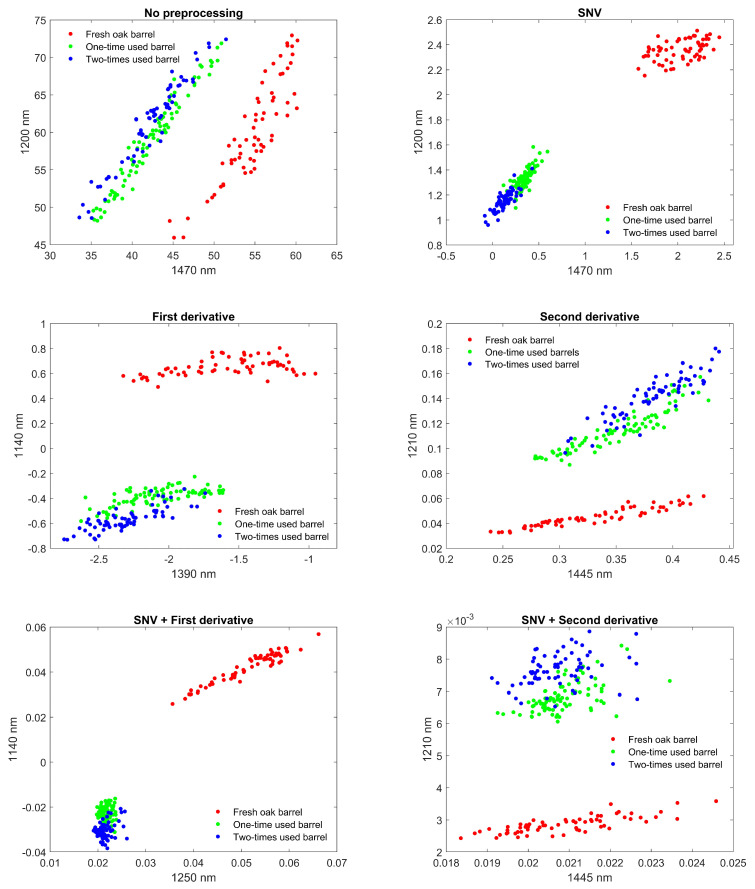
Scatter plot of the different measurement points on fresh, one–, and two–times wine refills marked with red, green, and blue dots, respectively. The scatter plot is extracted by reducing the reflectance spectra to a discrete set of two wavelengths. The wavelengths chosen correspond to characteristic absorption bands or maximum/minimum slopes in the derivative spectra. Clustering based on filling in 2 dimensions is improved by applying pre-processing techniques. SVN even has decent clustering in 1 dimension (1200 nm or 1470 nm) by projecting on either axis.

Reducing the dimensionality of the spectral data can potentially improve the classification performance, as well as computational time, and increase interpretability. The following techniques were implemented (each time for the different pre-processed spectra): (1) only keeping the NIR part of the spectrum (1050–1590 nm), since the UV/VIS part is mostly determined by the color of the wooden piece and has no direct physical relation to the chemical wine substances we are interested in; (2) only keeping a total of two wavelengths, corresponding to the absorption peaks or peaks of the derivative spectra; (3) Principal Component Analysis (PCA) with the number of principle components determined by cross-validation. When using only two specific wavelengths, scatter plots already show a clear separability between the three different classes (Figure 4).

Two different linear chemometric classification models were trained to separate the three different classes. The first technique that was used is Partial Least Squares Discriminant Analysis (PLS-DA). Only the NIR part of the spectra is used as input for the model. The classes (output) are represented by a binary vector such as [1 0 0], [0 1 0], [0 0 1] for fresh, one-, and two-times-used barrels, respectively. A threshold of 0.5 is used to convert the predicted continuous vectors back to a binary vector and class label. The number of used PLS components is determined by 5-fold cross-validation.

The second technique that was used is Linear Discriminant Analysis (LDA). A pooled, full covariance matrix was used in the model. LDA was applied on the NIR part of the spectra, on the two-dimensional scatter data, and also on the PCA transformed data (PCA-LDA). Due to the relatively low number of samples available, no separate hold-out test set was used. Standard 5-fold cross-validation is used to obtain a quasi-unbiased estimate of the classifier performance when no hyperparameters have to be chosen. Nested cross-validation, with five inner and five outer folds, are used to avoid a biased estimate when hyperparameters (number of PLS or PCA components) need to be tuned [30]. Ten repeats with a different random choice of the train and validation folds are performed to have a measure of the variance (and stability). The accuracy for the correct prediction of each three classes (diagonal of the confusion matrix) is used as an evaluation metric (Table 1).

The PLS-DA classifier, trained on the complete NIR range, clearly performed the best, independent of the used pre-processing. Both the samples of one-time wine filling and of two-times wine refills have been classified with >99% accuracy when doing repeated nested cross-validation. A stable model with little change in number of PLS components, and with low variability (change in accuracy) has been obtained, when considering different random train/test splits. The LDA classifier, when trained on the complete NIR range, performed slightly worse, although accuracy >93% was possible to obtain. Using the first derivative spectra has yielded the best results (>96%) in this case. The largest decrease in dimensionality was obtained by only using physical absorption characteristic wavelengths. Combined with LDA, an additional decrease in performance was observed, as expected. The most optimal results were now found when using the SNV pre-processing technique, with accuracy >88%. Moreover, significantly lower variability compared to using the whole NIR region was obtained. Lastly, when the LDA classifier was trained after doing dimensionality reduction and decorrelation using PCA, better results were obtained compared to only using absorption characteristics, but the results were in general worse compared to using PLS or LDA without PCA. In this case, the best results were achieved when using the first derivative spectra, both with and without doing SNV first, with accuracy >96%. However, the largest instabilities and variability were found with this model.

To summarize, the most optimal performance was achieved using PLS-DA on the whole NIR region, with the 2-dimensional (1200 nm, 1470 nm) LDA on the SNV pre-processed data a viable cost-effective alternative. In PLS-DA, the only operating (hyper) parameter that is crucial to obtaining a good classification performance is the number of principle components. Taking a too large number of components will lead to over-fitting, where the performance on new data will strongly deteriorate. On this data set, in the case of no pre-processing, over-fitting was found to occur at more than 30 components, with a steep drop in the validation set accuracy. This highlights the importance of strong cross-validation, to guarantee similar performance results on future measurements. LDA, on the other hand, only needs the mean and variance of the reflectance at the chosen wavelengths for future predictions. These model parameters can be updated later, when new measurements are available, to improve the performance over time.

### 3.2. Fluorescence Spectroscopy

As with the reflectance spectroscopy, multiple measurements were again performed on the inner surface of the three wooden pieces. A total of 65 measurements were performed on the fresh oak sample: 22 on the top, 22 on the middle, 21 on the bottom part; 84 measurements with the one-time-used oak barrel sample: 28 on the top, 28 on the middle, 28 on the bottom segment; and, finally, 69 measurements on the two-times-refilled oak barrel sample: 23 on the top, 23 on the middle, 23 on the bottom part. All measurement points were again distributed equivalently over the horizontal section of the inside of the wood.

Although most pre-processing techniques discussed before are often used for reflectance spectroscopy data, they have also been applied in this study to the fluorescence data to determine possible improvements. The following techniques are again implemented as before: (1) Standard Normal Variate transformation; (2) 1st derivative using a Savitsky–Golay filter of order 2 and length of 11 points; (3) 2nd derivative using a Savitsky–Golay filter of order 2 and length of 11 points. By verifying the classification performances, it was found that no further improvements could be seen from combining SNV with a derivative spectra.

**Figure 5:**
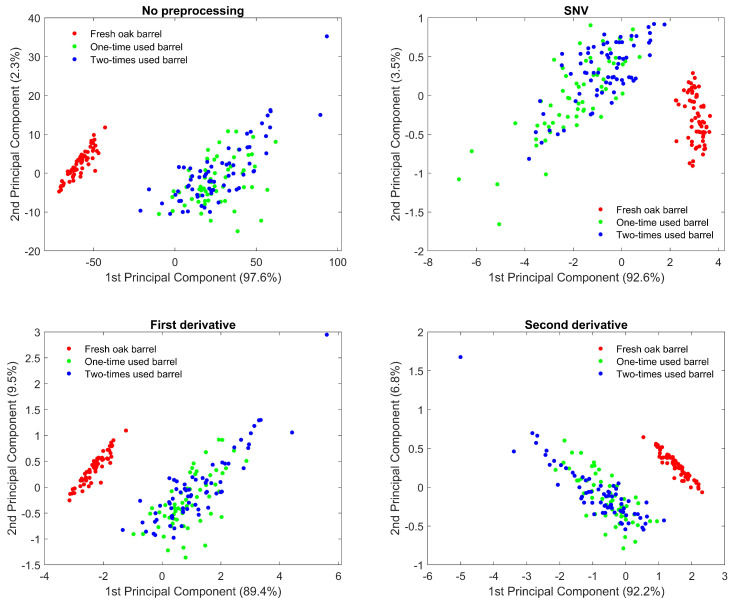
Scatter plot of the first 2 principal components using PCA. The amount of variance explained is written in between brackets on the axis labels. The separation between one– and two–times–used barrels was not possible using the 2 first components, as could already be expected from the variable and overlapping raw fluorescence data.

Next to using the emission spectrum at 374–464 nm spectral range, Principal Component Analysis (PCA) can be used to reduce the dimensionality and decorrelate the data. The number of principle components are determined by cross-validation. To visually inspect the samples, the first two principal components can be retained, showing a separation between fresh and one- and two-times-used clusters only (Figure 5). The use of the excitation peaks alone did not yield any significant differences between the different classes.

As before, two linear chemometric classification models were trained to separate the three different classes. Partial Least Squares Discriminant Analysis was applied on the whole emission spectra without dimensionality reduction. The same parameters as in reflectance spectroscopy were used. The number of PLS components are determined by 5-fold cross-validation. Linear Discriminant Analysis was applied once on the whole emission spectra, and once on the PCA transformed data (PCA-LDA). A pooled, full covariance matrix is used. The number of used PCA components is determined by 5-fold cross-validation.

Repeated 5-fold (nested) cross-validation was again used to determine the accuracy for classifying the three different classes (Table 2).

Since fluorescence is a phenomenon with intrinsically larger variance, which could already be seen from the raw measurements, it has been expected that classification would not be straightforward. The LDA classifier, trained on the complete emission spectra, has not resulted in any possibility to distinguish between the three samples with accuracy < 40%. It is clear that a projection into a different space, using PLS or PCA is needed. By using the PLS-DA or PCA-LDA classifier, on the other hand, better performances have been obtained. Compared to the results based on the reflectance spectroscopy, however, the accuracy have still been significantly lower. Using fluorescence, it was found that samples with one-time filling were classified with a larger accuracy compared to samples with two-times refills, which were often misclassified as having only a single filling.

Traditional pre-processing techniques have been demonstrated not to have a significant positive impact on the performances obtained. The best classification results have been obtained when using the LDA classifier after transforming the raw spectra (no pre-processing) using PCA. The samples with one-time filling have been classified with +/−87% accuracy, while the samples with two-times refills have an +/−70% accuracy, respectively. The misclassified samples of one-time filling have been recognized as two-times refills, and vice-versa. The model is relatively stable and demonstrated a variability of about 0.7% standard error depending on the train/test split.

### 3.3. Raman Spectroscopy

Multiple measurements have also been performed on the inner surface of the three wooden pieces by using Raman spectroscopy [31]. During Raman spectroscopy measurements, the barrel stave was cut into smaller pieces to fit under the microscope. A total of 30 sampling points were taken for each of the three different classes because Raman measurements are more time-consuming than reflectance or fluorescence spectroscopy measurements. A longer acquisition time was needed to obtain distinguishable Raman peaks. Moreover, the Raman signal depends on the laser spot size at the focal point. Due to the high surface roughness of the oakwood, the measured Raman intensity highly depends on the position of the measurement. The results of the measurements can thus be significantly different even for different points of the same wooden pieces. This, in turn, makes the classification very difficult.

**Figure 6:**
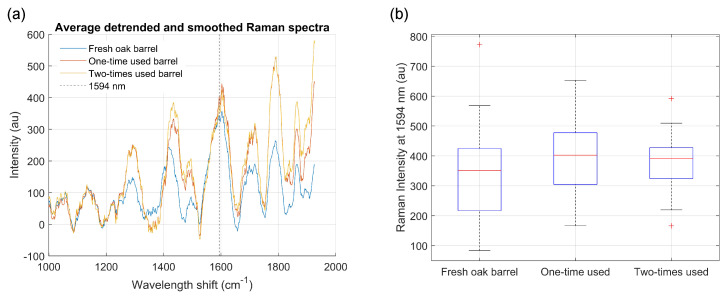
(**a**) Average detrended and smoothed Raman signals of the three oak samples. (**b**) Box-plot of the Raman signal at 1594 nm, which is the characteristic tannin peak. Large variability is present due to the localized measurement technique, influenced by the inhomogeneous wood itself, showing that the different classes are inherently not separable.

To remove the large background fluorescence signal that was present on the raw Raman signals (see Figure 3c), detrending with the intelligent fitting option present in Renishaw WiRE software was applied. Next, the signal was smoothed by a moving average filter with 25 points average (Figure 6a). A large variability has been observed on the Raman signal for different points from the same oak piece, larger than a noticeable difference between different oak samples from one- and two-times-used barrels. This large amount of overlap is also clearly visible on the specific Raman peak of tannin (Figure 6b).

Here, as in the previous two cases, the SNV, 1st derivative signal, and 2nd derivative signals were calculated to potentially improve the classification performance. However, as expected, they were not able to do so. In addition, applying PCA did not improve the separation between the different samples.

Finally, neither LDA nor PLS-DA were able to achieve any decent classification performance. This showed that with this measurement technique, the classes were inherently not separable. The main reason was the additional influence of the inhomogeneous wood, which varied based on the measurement location. The influence of tannin on the Raman spectra was not strong enough to see any difference between fresh, one-, and two-times-used oakwood samples.

## 4. Conclusions

Three different spectroscopic measurement techniques have been applied on fresh, one-, and two-times-used barrel samples from French oak. The presence of chemical compounds, such as lignin and tannin, present in oakwood that give the wine its specific taste, is examined with the mentioned spectroscopic techniques by studying their relative quantity on the inner surface of an oak barrel used in winemaking. Standard chemometric processing techniques, including pre-processing (none, SNV, 1st derivative, 2nd derivative), dimensionality reduction (none, PCA, physical wavelengths) and classifier training (LDA, PLS-DA), have been implemented. The accuracy of classifying the samples to their correct number of wine refills has been evaluated using repeated nested cross-validation.

Reflectance spectroscopy has clearly demonstrated the best classification performance. The highest accuracy was obtained by a PLS-DA model, with the number of components carefully chosen by a grid search and cross-validation. Using the whole NIR spectra without any pre-processing, the PLS-DA model has been able to classify almost all samples (>99%) correctly. The LDA classifier has been a viable alternative but more depending on the choice of pre-processing method. Using SNV, a decent performance (>88%) can be obtained using reflectance at two absorption peaks. First derivative spectra, combined with a PCA transformation, has also shown good accuracy (>96%).

Fluorescence spectroscopy has demonstrated a significantly lower performance. Due to the larger variation in the natural autofluorescence signals, classification has been more difficult. The best performance (>70%) was obtained on the raw spectra when using PCA in combination with LDA. Using PLS-DA on the raw spectra yielded slightly lower performances, but still significantly larger compared to LDA without PCA. Using only the emission peak signals was not sufficient to distinguish between one-time and two-times refills.

Finally, the used Raman spectroscopy technique cannot be considered to classify different samples. Due to the variety of localized measurements, the influence of the oakwood itself played an important role in the obtained signals. The influence of tannin, the main marker of wine usage, was not strong enough to be visible across the different samples.

As a conclusion of these benchmarking techniques, it is important to note that, ideally, a large enough external test set consisting of several other oak samples from fresh, one-, and two-times-used barrels are needed to fully validate the obtained models. Due to the limited number of barrel samples, external validation has not been fully possible. More samples from different times of refills will improve the classification algorithms as well. However, in this feasibility study, only a single oakwood piece was available per wine refill, thus requiring multiple measurements along the same piece to simulate the effect of having multiple pieces.

Based on the outcome of various signal processing algorithms, reflectance spectroscopy can be the technique of choice with appropriate data analyzing methods. Several prototype portable biosensing devices working on this principle are available, such as the one from [32]. The latter is already used in the food industry to determine fruit ripeness and could be adapted for wine barrel usage instead. Nevertheless, the difference in instrumental settings and resolution can yield experimental data differences. Hence, for this purpose, a set of new measurements on oakwood barrel pieces may be required to implement data processing algorithms and define appropriate parameters for accurate classification. Nonetheless, to our knowledge, this is the first work that reports the use of various spectroscopic techniques in combination with data processing algorithms to classify oak barrels in winemaking to unveil their reusability. The outcome of this study paves a path for further development of biosensing techniques, based on reflectance spectroscopy, to reveal the effectiveness of oak barrels for wine ageing after several refills. This non-invasive approach to oak barrels qualification can have a high economic impact on the wine industry.

## Figures and Tables

**Table 1:** Classification results based on reflectance spectroscopy. The optimal number of PLS or PCA components is denoted in brackets. The validation accuracy is given in the table, obtained by using repeated (nested) 5-fold cross-validation. The error is the standard error of the mean obtained by the 10 different repeats.

Pre-Proc.	Dim. Red.	Classifier	Acc. Fresh Oak (%)	Acc. One Time Used (%)	Acc. Two Times Used (%)
	NIR	PLS-DA (15)	100.0 ± 0.0	99.8 ± 0.2	100.0 ± 0.0
	NIR	LDA	100.0 ± 0.0	95.6 ± 0.6	95.1 ± 1.1
None	Absorption	LDA	100.0 ± 0.0	88.2 ± 0.4	69.0 ± 0.8
	PCA (10)	LDA	100.0 ± 0.0	89.6 ± 1.6	84.6 ± 2.4
	NIR	PLS-DA (18)	100.0 ± 0.0	100.0 ± 0.0	99.1 ± 0.3
	NIR	LDA	99.8 ± 0.2	94.9 ± 0.5	94.9 ± 1.0
SNV	Absorption	LDA	100.0 ± 0.0	88.9 ± 0.2	88.2 ± 0.2
	PCA (25)	LDA	100.0 ± 0.0	90.4 ± 1.7	91.0 ± 1.4
	NIR	PLS-DA (22)	100.0 ± 0.0	99.2 ± 0.3	99.6 ± 0.2
	NIR	LDA	100.0 ± 0.0	97.1 ± 0.8	96.5 ± 0.8
1st der.	Absorption	LDA	100.0 ± 0.0	89.2 ± 0.1	82.4 ± 0.0
	PCA (26)	LDA	100.0 ± 0.0	96.3 ± 0.6	96.0 ± 0.5
	NIR	PLS-DA (22)	100.0 ± 0.0	99.3 ± 0.4	99.7 ± 0.2
	NIR	LDA	100.0 ± 0.0	97.3 ± 0.7	95.6 ± 0.8
2nd der.	Absorption	LDA	100.0 ± 0.0	85.2 ± 0.3	80.4 ± 0.4
	PCA (30)	LDA	100.0 ± 0.0	94.5 ± 1.2	93.5 ± 1.3
	NIR	PLS-DA (22)	100.0 ± 0.0	99.0 ± 0.2	98.2 ± 0.4
	NIR	LDA	100.0 ± 0.0	94.9 ± 0.6	93.8 ± 0.9
SNV + 1st der.	Absorption	LDA	100.0 ± 0.0	89.0 ± 0.4	88.2 ± 0.0
	PCA (40)	LDA	100.0 ± 0.0	96.5 ± 0.7	97.1 ± 0.4
	NIR	PLS-DA (35)	100.0 ± 0.0	99.5 ± 0.5	97.4 ± 0.7
	NIR	LDA	100.0 ± 0.0	96.7 ± 0.4	96.5 ± 0.7
SNV + 2nd der.	Absorption	LDA	100.0 ± 0.0	87.4 ± 0.2	82.9 ± 0.3
	PCA (34)	LDA	100.0 ± 0.0	89.3 ± 0.9	92.9 ± 0.7

**Table 2:** Classification results using fluorescence spectroscopy. The optimal number of PLS or PCA components is denoted in brackets. The validation accuracy is given in the table, obtained by using repeated (nested) 5-fold cross-validation. The error is the standard error of the mean obtained by the 10 different repeats.

Pre-Proc.	Dim. Red.	Classifier	Acc. Fresh Oak (%)	Acc. One Time Used (%)	Acc. Two Times Used (%)
	None	PLS-DA (7)	100.0 ± 0.0	84.2 ± 0.5	67.9 ± 0.5
None	None	LDA	79.4 ± 2.4	31.5 ± 2.3	35.7 ± 2.1
	PCA (5)	LDA	100.0 ± 0.0	87.1 ± 0.7	70.3 ± 0.7
	None	PLS-DA (7)	100.0 ± 0.0	77.9 ± 0.9	67.2 ± 0.4
SNV	None	LDA	78.9 ± 2.5	33.5 ± 1.8	34.1 ± 1.8
	PCA (8)	LDA	100.0 ± 0.0	77.9 ± 1.2	69.7 ± 0.7
	None	PLS-DA (7)	100.0 ± 0.0	81.5 ± 0.8	67.5 ± 0.8
1st der.	None	LDA	85.1 ± 1.7	38.3 ± 1.9	32.7 ± 1.6
	PCA (8)	LDA	100.0 ± 0.0	80.9 ± 0.5	68.8 ± 0.6
	None	PLS-DA (7)	100.0 ± 0.0	78.9 ± 1.1	64.5 ± 0.7
2nd der.	None	LDA	83.8 ± 1.3	36.9 ± 1.9	35.8 ± 2.7
	PCA (20)	LDA	100.0 ± 0.0	57.2 ± 1.9	46.2 ± 2.3

## Data Availability

The data that supports this study are available from corresponding authors upon reasonable request.
